# Machine learning-driven prognostic prediction model for composite small cell lung cancer: identifying risk factors with network tools and validation using SEER data and external cohorts

**DOI:** 10.3389/fonc.2025.1633635

**Published:** 2025-07-23

**Authors:** Fei Li, Mengfan Zhao, Linlin Cao, Shuai Qie

**Affiliations:** ^1^ School of Nursing, Hebei University, Baoding, China; ^2^ Neurology Intensive Care Unit, Affiliated Hospital of Hebei University, Baoding, China; ^3^ Department of Radiation Oncology, Affiliated Hospital of Hebei University, Baoding, China

**Keywords:** SEER (surveillance epidemiology and end results) database, c-SCLC, SHAP (Shapley additive explanation), machine learning, composite small cell lung cancer

## Abstract

**Background:**

Lung cancer continues to be the primary cause of cancer-related mortality globally, with combined small cell lung carcinoma (C-SCLC) constituting a relatively uncommon yet highly aggressive subset of this disease. Despite its clinical significance, limited efforts have been made to develop survival prediction models tailored to the clinical characteristics of C-SCLC patients. Additionally, the interpretability of existing models remains limited.

**Methods:**

This study aimed to develop and validate an interpretable machine learning model for predicting survival outcomes in C-SCLC patients using clinical data from the SEER database and external validation with Chinese patient cohorts. Initially, we employed the Cox proportional hazards model for rigorous variable selection. Subsequently, through 10-fold cross-validation and grid search for optimal parameters, we selected the XGBoost model as the best-performing one among four candidates. Furthermore, we enhanced the model’s interpretability by incorporating the SHapley Additive exPlanations (SHAP) method, which helped us understand the contribution of each variable within the model.

**Results:**

We constructed a predictive model using data from 1,230 SEER patients and validated it externally with data from 154 Chinese patients. The XGBoost model demonstrated excellent performance in predicting survival outcomes at 1-year, 3-year, and 5-year. The AUC values for the external validation cohort were 0.849, 0.830, and 0.811, respectively. SHAP analysis revealed that N stage, T stage, radiotherapy, surgery, and gender are key factors influencing the ML model’s predictions. To enhance clinical utility, we have developed an interpretable web-based tool to predict patients’ 1-year survival probability.

**Conclusion:**

The XGBoost model, integrating demographic and clinical factors of C-SCLC patients, demonstrated excellent predictive performance. Our web-based prediction tool will promote the development of personalized treatment strategies and optimize clinical decision-making.

## 1 Introduction

The highest incidence rate among all types of cancer is due to lung cancer, which is the leading cause of cancer mortality worldwide (1). Combined- small cell lung cancer(C-SCLC) is a relatively rare subtype of lung cancer (2, 3), with an overall five-year survival rate of 12.4% (4). According to the World Health Organization’s (WHO) classification of tumors, C-SCLC is defined as a mixture of small cell lung cancer and any type of non-small cell lung histological type (5, 6). This definition emphasizes the histological specificity of C-SCLC, which encapsulates the highly aggressive and rapid growth characteristics of small cell lung cancer (SCLC) as well as the components of non-small cell carcinoma (7). The complexity of C-SCLC makes it particularly challenging to diagnose, treat, and predict survival. This study is dedicated to developing a prediction model based on explainable Machine Learning (ML) for accurately predicting the survival outcomes of patients with C-SCLC. The clinical application potential of this model is immense, as it can provide doctors and patients with more objective and accurate survival predictions, thereby assisting in the formulation of personalized treatment strategies and optimizing clinical decision-making processes.

However, significant issues still exist in the field of survival prediction for C-SCLC. Despite the increasing application of ML technology in the medical field in recent years, research on constructing survival prediction models based on patient clinical characteristics for this relatively rare subtype of lung cancer remains scarce. Furthermore, even though ML models exhibit impressive predictive performance, their “black box” nature (8), the difficulty in directly explaining the mechanism behind the model’s predictions—limits their widespread application in clinical decision-making.

The healthcare sector has witnessed substantial growth in the utilization of ML technology in recent years, demonstrating impressive proficiency in data processing and pattern recognition (9–11). Concurrently, clinical researchers have been diligently employing a spectrum of ML technologies to furnish evidence-based recommendations for cancer management. As the tumor with the highest incidence, lung cancer understandably commands substantial attention, and discernible progress has been achieved in its immunotherapy, patient screening, and radiomics (12–14), with relatively few studies investigating survival prediction models for C-SCLC patients. Additionally, although some research has made progress in exploring the interpretability of ML models, studies on the interpretability of survival prediction models remain limited.

In the study by Yang et al. (15) the clinical features of C-SCLC patients were integrated to successfully construct a high-performance survival prediction model (15). In our study, we extracted more detailed clinical characteristics of patients from the SEER database. We then compared the performance of four machine learning algorithms and identified a model with the most outstanding performance. Finally, we externally validated this model using data from 154 Chinese C-SCLC patients, demonstrating the model’s predictive accuracy across different racial groups, and developed a web-based tool for use by medical practitioners worldwide. ML models often lack interpretability, which makes it difficult for physicians to understand and trust their predictions. This issue limits their practical application in clinical decision-making. To overcome the “black-box” nature of these models, we used SHAP (SHapley Additive exPlanations) technology to elucidate the predictions of the ML model and provide visual representations of the impact of individual variables.

The model not only has more robust predictive performance but is also accessible to physicians via an online platform. Moreover, it provides intuitive and understandable explanations of the prediction results, thereby helping them to formulate more precise treatment strategies. This study is expected to provide new insights and methods for clinical decision-making in C-SCLC patients and promote the further development of machine learning technology in oncological clinical research.

## 2 Materials and methods

### 2.1 Study data

**Figure 1:**
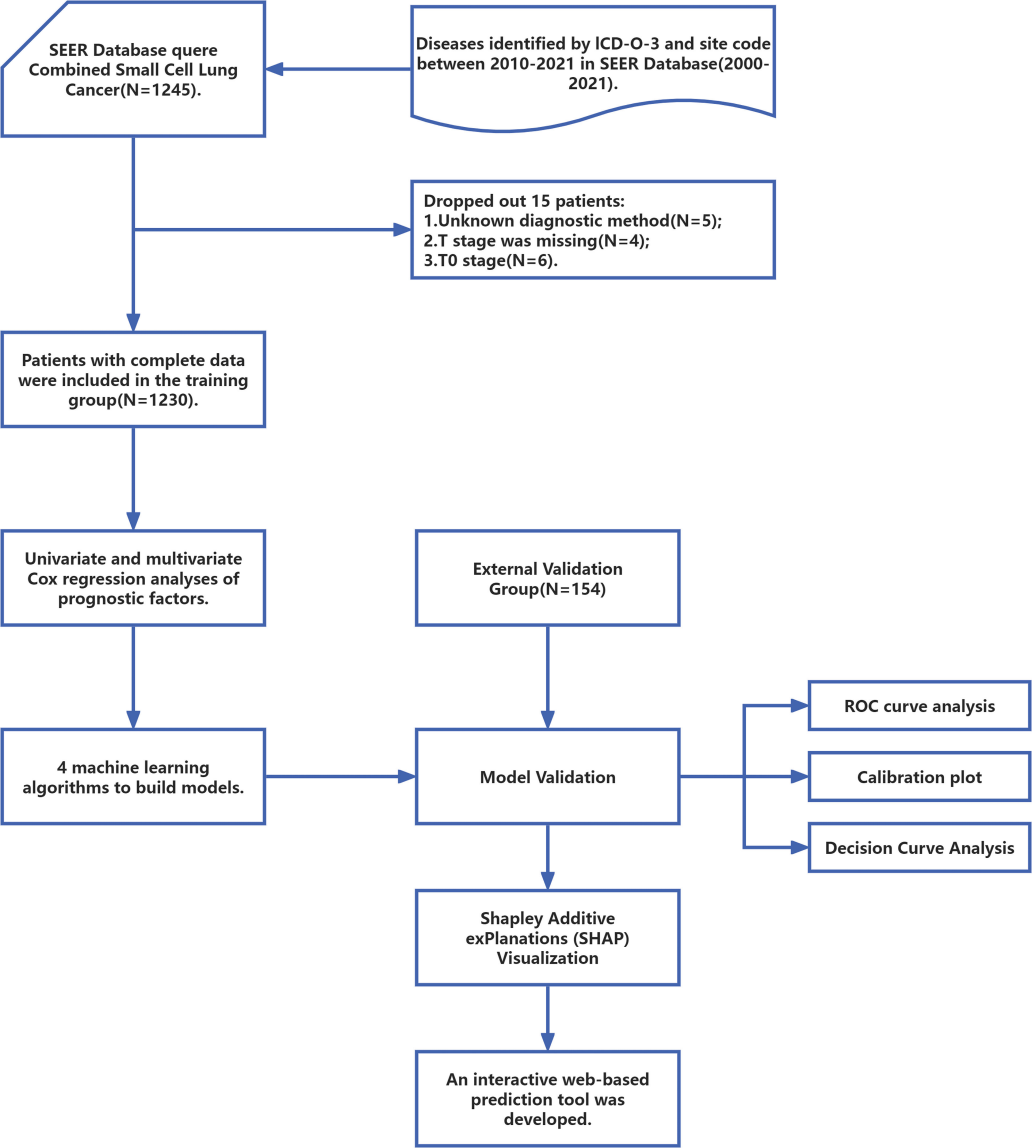
The workflow diagram for study design and patient screening. SEER, The Surveillance, Epidemiology, and End Results; ICD-O-3, International Classification of Diseases for Oncology; ROC, Receiver Operating Characteristic.

SEER Stat software (SEER*Stat, v8.4.0.1) was used to extract clinical data from 2010 to 2021 from the SEER database (Incidence - SEER Research Data, 17 Registries, Nov 2023 Sub (2000-2021)). To ensure the integrity of the data and the accuracy of the analysis, this study conducted data preprocessing by removing records with missing values. The screening and exclusion criteria were as follows:(1) The primary sites of the tumor must be the lung and bronchus, coded as C34.0, C34.1, C34.2, C34.3, C34.8, and C34.9 according to the International Classification of Disease for Oncology, Third Edition (ICD-O-3) topography;(2) The tumor must be pathologically confirmed as combined C-SCLC, coded as 8045/3 in the ICD-O-3.(3) Patients will be excluded if their diagnostic methods are unknown or if they lack complete clinical information, treatment data, and survival data. A total of 1230 patients with C-SCLC were ultimately recruited from the SEER database. Additionally, patients diagnosed and treated for C-SCLC at the Affiliated Hospital of Hebei University between 2000 and 2021 were screened. Patients without complete clinical characteristic data and survival information were excluded. Consequently, 154 patients were ultimately included in the external validation group. Due to the research design, this study was approved by the Ethics Committee of the Affiliated Hospital of Hebei University (HDFT-LL-2022-075) and was conducted in accordance with the principles outlined in the Declaration of Helsinki. **Figure 1** provides a clear depiction of the entire workflow of the proposed system.

### 2.2 Data extraction and end point

Data variables required for analysis were extracted, including age, sex, race, marital status, primary site, T stage, N stage, bone metastasis, brain metastasis, liver metastasis, lung metastasis, metastasis to other organs, surgery, radiotherapy, chemotherapy, survival months, and vital status. The primary endpoint of this study was overall survival (OS). Survival time was calculated from the date of diagnosis to the date of the last follow-up, or until the date of death due to any cause.

### 2.3 Statistical analysis

Statistical analyses were performed using R version 4.2.3 and python version 3.11.4. The cut-off points for continuous variables were ascertained using X-tile software (version 3.6.1) and subsequently transformed into categorical variables. A descriptive analysis of the clinical baseline characteristics of the enrolled patients was performed, and the chi-square test was employed to assess differences in features between the training and validation groups. We conducted univariate Cox proportional hazards regression analysis using the data from the training cohort of patients to identify variables that are statistically significantly associated with patient survival outcomes. Subsequently, these significant variables were included in a multivariate Cox proportional hazards regression model. All statistical tests were two-tailed, with a significance level set at p < 0.05.

### 2.4 Model construction and evaluation metrics

Based on the selected variables, we proceeded to incorporate them into four distinct ML models: Logistic Regression (LR), Support Vector Machine (SVM), Random Forest (RF), and eXtreme Gradient Boosting (XGBoost). Grid search methods were used to determine the best performing hyperparameters for the four models. We used 10-fold cross-validation to determine the best performance. To evaluate the performance of the four models, we compared their AUC, accuracy, sensitivity, and specificity in both the validation and training sets. The hyperparameters selected for the final XGBoost model were as follows: colsample_bytree: 1, learning_rate: 0.3, max_depth: 8, min_child_weight: 4, n_estimators: 20, reg_lambda: 0.5, subsample: 1. The calibration curve comparing the mean predicted survival rate with the actual survival rate was used to assess the calibration of the model. Decision curve analysis (DCA) was performed by calculating the net benefits for a range of threshold probabilities to assess the clinical utility of the model (16).

### 2.5 SHAP explainability analysis

We employed SHAP to interpret the most effective predictive model. For tree-based models, TreeExplainer was employed due to its computational efficiency and precise SHAP value calculations (17). In contrast, KernelExplainer—a model-agnostic alternative—was applied to other model types, though it is slower and less precise (18). The choice of explainer depended on the model architecture and the trade-off between speed and flexibility. To rank features by importance, we calculated their mean absolute SHAP values. This approach not only quantifies the global contribution of each feature but also reveals how individual features influence specific predictions.

## 3 Results

### 3.1 Baseline characteristics

From the SEER database, a total of 1,230 patients were selected as the training cohort. Among them, 60% of the patients were ≤71 years old, 26% were between 72-79 years old, and 14% were ≥80 years old. The gender distribution was 56% male and 44% female. Regarding the primary tumor location, 52% of the patients had their primary site in the main bronchus, 30% in the upper right lobe, 3.5% in the middle right lobe, 15% in the lower right lobe, 26% in the upper left lobe, 11% in the lower left lobe, and 11% in unspecified locations. Additionally, a validation cohort of 154 patients was constituted, with 64%, 21%, and 14% of the patients belonging to the aforementioned age groups, respectively; 68% were male and 32% were female. Detailed demographic and clinical characteristics are shown in **Table 1**.

**Table 1:** Demographics and characteristics of patients in training and testing group.

Group				
Variable	Overall N=1384^1^	Validation cohort from China N = 154^1^	Training cohort from SEER database N = 1230^1^	P-value^2^
Sex n (%)				0.002
Female	612 (44)	50 (32)	562 (46)	
Male	772 (56)	104 (68)	668 (54)	
Age n (%)				0.409
≤71	832 (60)	99 (64)	733 (60)	
72-79	357 (26)	33 (21)	324 (26)	
≥80	195 (14)	22 (14)	173 (14)	
Marital status n (%)				0.158
Married	1104 (80)	114 (74)	990 (80)	
Unmarried	216 (16)	30 (19)	186 (15)	
Unknown	64 (4.6)	10 (6.5)	54 (4.4)	
Primary Site n (%)				0.335
Main bronchus	72 (5.2)	12 (7.8)	60 (4.9)	
Right upper lobe	403 (29)	46 (30)	357 (29)	
Right middle lobe	48 (3.5)	5 (3.2)	43 (3.5)	
Right lower lobe	203 (15)	18 (12)	185 (15)	
Left upper lobe	358 (26)	38 (25)	320 (26)	
Left lower lobe	154 (11)	13 (8.4)	141 (11)	
Unspecific	146 (11)	22 (14)	124 (10)	
T n (%)				0.096
T1	322 (23)	37 (24)	285 (23)	
T2	355 (26)	28 (18)	327 (27)	
T3	249 (18)	25 (16)	224 (18)	
T4	340 (25)	48 (31)	292 (24)	
TX	118 (8.5)	16 (10)	102 (8.3)	
N n (%)				0.025
N0	465 (34)	36 (23)	429 (35)	
N1	139 (10)	12 (7.8)	127 (10)	
N2	500 (36)	67 (44)	433 (35)	
N3	222 (16)	31 (20)	191 (16)	
NX	58 (4.2)	8 (5.2)	50 (4.1)	
Bone metastasis n (%)				0.350
No/Unknown	1168 (84)	126 (82)	1042 (85)	
Yes	216 (16)	28 (18)	188 (15)	
Brain metastasis n (%)				0.384
No/Unknown	1191 (86)	129 (84)	1062 (86)	
Yes	193 (14)	25 (16)	168 (14)	
Liver metastasis n (%)				0.035
No/Unknown	1168 (84)	121 (79)	1047 (85)	
Yes	216 (16)	33 (21)	183 (15)	
Lung metastasis n (%)				0.958
No/Unknown	1224 (88)	136 (88)	1088 (88)	
Yes	160 (12)	18 (12)	142 (12)	
Others metastasis n (%)				0.391
No/Unknown	1249 (90)	136 (88)	1113 (90)	
Yes	135 (9.8)	18 (12)	117 (9.5)	
Surgery n (%)				0.013
No/Unknown	1027 (74)	127 (82)	900 (73)	
Yes	357 (26)	27 (18)	330 (27)	
Radiatherapy n (%)				0.194
No/Unknown	751 (54)	76 (49)	675 (55)	
Yes	633 (46)	78 (51)	555 (45)	
Chemotherapy n (%)				0.009
No/Unknown	462 (33)	37 (24)	425 (35)	
Yes	922 (67)	117 (76)	805 (65)	

^1^Median (IQR) or Frequency (%).

^2^Pearson’s Chi-squared test.

### 3.2 Prognosis analysis of C-SCLC

To identify prognostic variables, univariate and multivariate Cox regression analyses of overall survival (OS) were performed for patients with C-SCLC from the SEER database. The following variables were associated with OS: gender, age (≤71 years, 72-79 years, ≥80 years), T stage, N stage, surgery, chemotherapy, radiotherapy, bone metastasis, brain metastasis, liver metastasis and other distant metastases were correlated with patient survival outcomes (**Table 2**, *P<*0.05).

**Table 2:** Univariate and multivariate Cox analysis of prognostic factors.

Characteristics	Uni-HR	Uni-CI	Uni-P	Multi-HR	Multi-CI	Multi-P
Sex	1.166	1.004-1.355	0.044	1.208	1.035-1.409	0.016
Age	1.235	1.116-1.368	<0.001	1.258	1.131-1.398	<0.001
Marital status	0.909	0.789-1.047	0.186			
Race	0.972	0.84-1.126	0.708			
Primary Site	1.079	1.032-1.127	0.001			
T	1.315	1.242-1.392	<0.001	1.122	1.051-1.198	0.001
N	1.387	1.304-1.476	<0.001	1.226	1.14-1.319	<0.001
Bone metastasis	2.353	1.923-2.879	<0.001	1.398	1.114-1.756	0.004
Brain metastasis	1.782	1.436-2.212	<0.001	1.493	1.179-1.89	0.001
Liver metastasis	2.57	2.102-3.143	<0.001	1.515	1.211-1.895	<0.001
Lung metastasis	2.181	1.76-2.703	<0.001	1.159	0.918-1.462	0.215
Others metastasis	1.585	1.239-2.028	<0.001	1.331	1.019-1.739	0.036
Surgery	0.362	0.299-0.437	<0.001	0.415	0.328-0.526	<0.001
Radiatherapy	0.863	0.743-1.003	0.054	0.659	0.552-0.787	<0.001
Chemotherapy	0.705	0.603-0.824	<0.001	0.5	0.418-0.598	<0.001

### 3.3 Performance comparison and Identification of the final model

**Figure 2:**
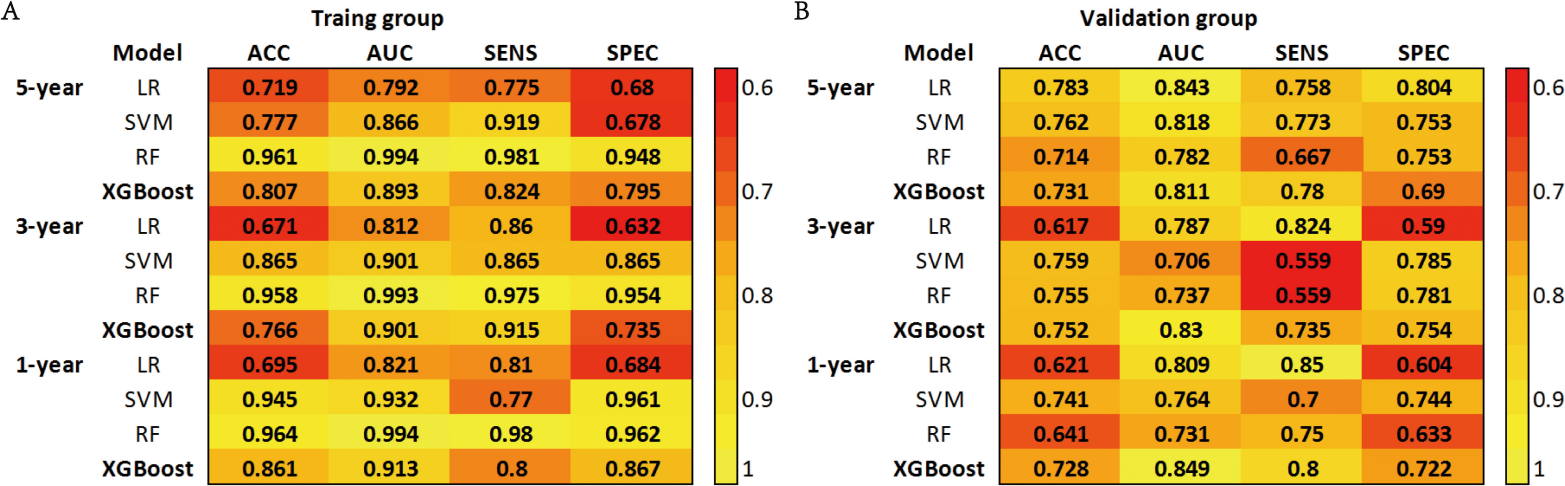
**(A)** Prediction performance of four models in the training group. **(B)** Prediction performance of four models in the validation group. ACC, Accuracy; AUC, Area Under the Curve; SENS, Sensitivity; SPEC, Specificity; LR, Logistic Regression; SVM, Support Vector Machine; RF, Random Forest; XGBoost, Extreme Gradient Boosting.

Based on the aforementioned results, we incorporated 11 independent risk factors, including gender, age (≤71 years, 72-79 years, ≥80 years), T stage, N stage, surgery, chemotherapy, radiotherapy, bone metastasis, brain metastasis, liver metastasis and other distant metastases, into four distinct machine learning models to evaluate their predictive performance in both the training and validation groups. Utilizing a 10-fold cross-validation method, the predictive capabilities of the four ML models were thoroughly evaluated (**Figure 2**).

**Figure 3:**
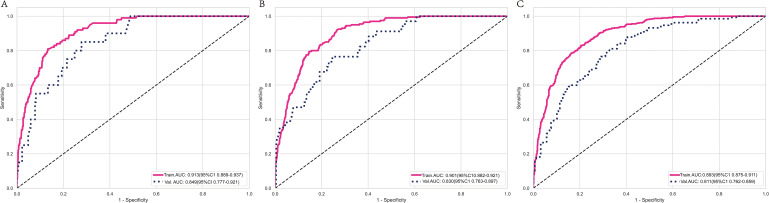
ROC curves for the XGBoost model’s survival predictions in the training and validation group at 1-year **(A)**, 3-year **(B)**, and 5-year **(C)**. ROC curves for the XGBoost model show that the model performs excellently on both the training and validation sets.

The SVM model demonstrated AUC values of 0.932, 0.901, and 0.866 in the training groups for predicting 1-year, 3-year, and 5-year overall survival (OS), respectively. However, in the validation groups, the AUC values decreased to 0.764, 0.706, and 0.818. This indicates overfitting of the model. The RF model exhibited a similar situation, with excellent performance in the training groups but a significant drop in AUC values in the validation groups. The XGBoost model performed well in predicting 1-year, 3-year, and 5-year survival in C-SCLC patients. The training group AUCs were 0.913 (95% CI: 0.889 - 0.937), 0.901 (95% CI: 0.882 - 0.921), and 0.893 (95% CI: 0.875 - 0.911). The validation group AUCs were 0.849 (95% CI: 0.777 - 0.921), 0.830 (95% CI: 0.763 - 0.897), and 0.811 (95% CI: 0.762 - 0.859) (**Figure 3**). The XGBoost model demonstrates exceptional performance across accuracy, precision, and sensitivity metrics, which substantiates its superior generalization capability over the other three ML models. Ultimately, the XGBoost model was selected as the final model and underwent a more comprehensive evaluation.

**Figure 4:**
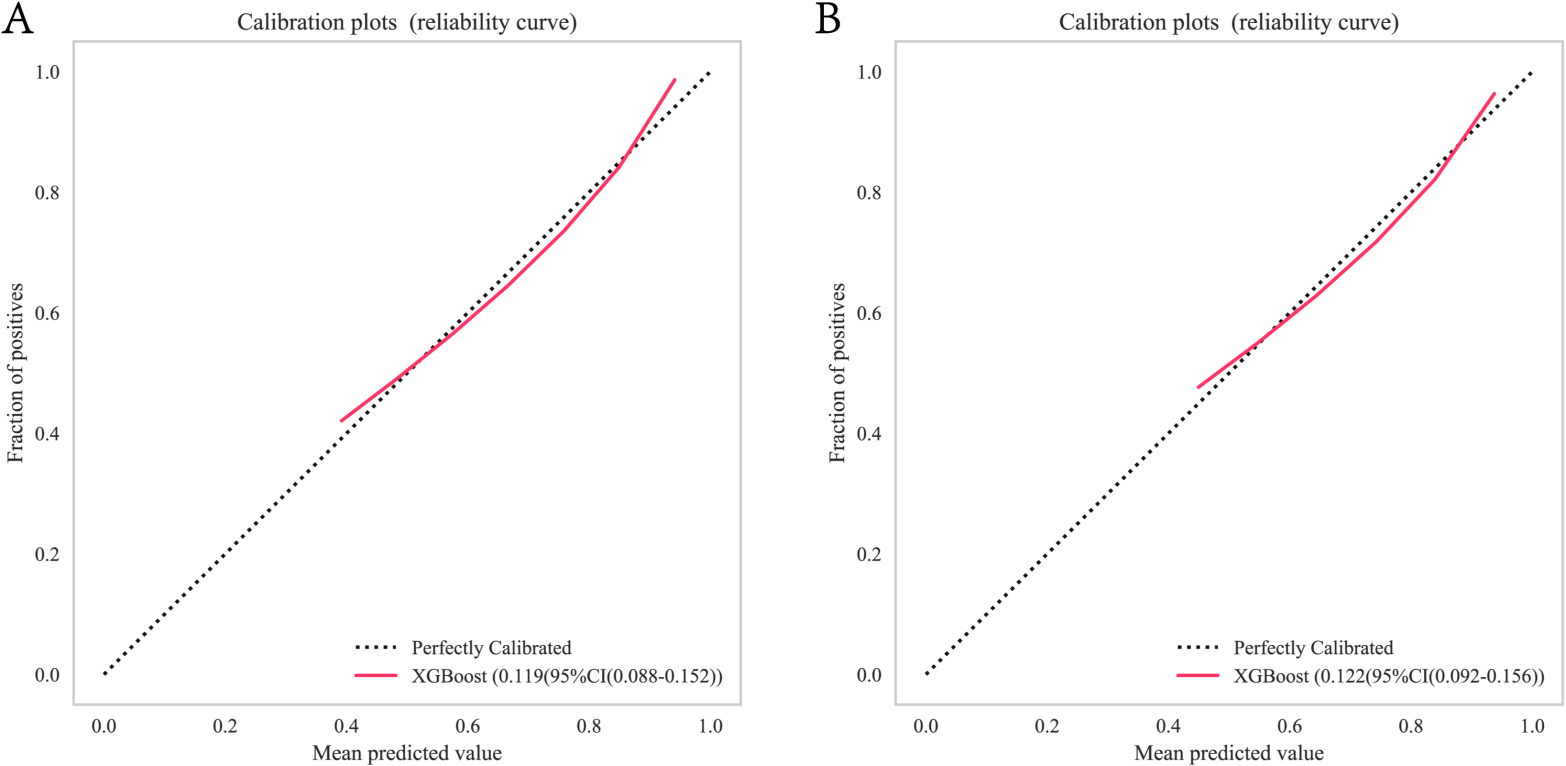
XGBoost model calibration curves for predicting 1-year OS: **(A)** Calibration curves for the training group. **(B)** Calibration curves for the validation group. OS, overall survival. Calibration curves for the XGBoost model illustrating the agreement between predicted and observed 1-year OS in training and validation cohorts. OS, overall survival. XGBoost, Extreme Gradient Boosting.

**Figure 5:**
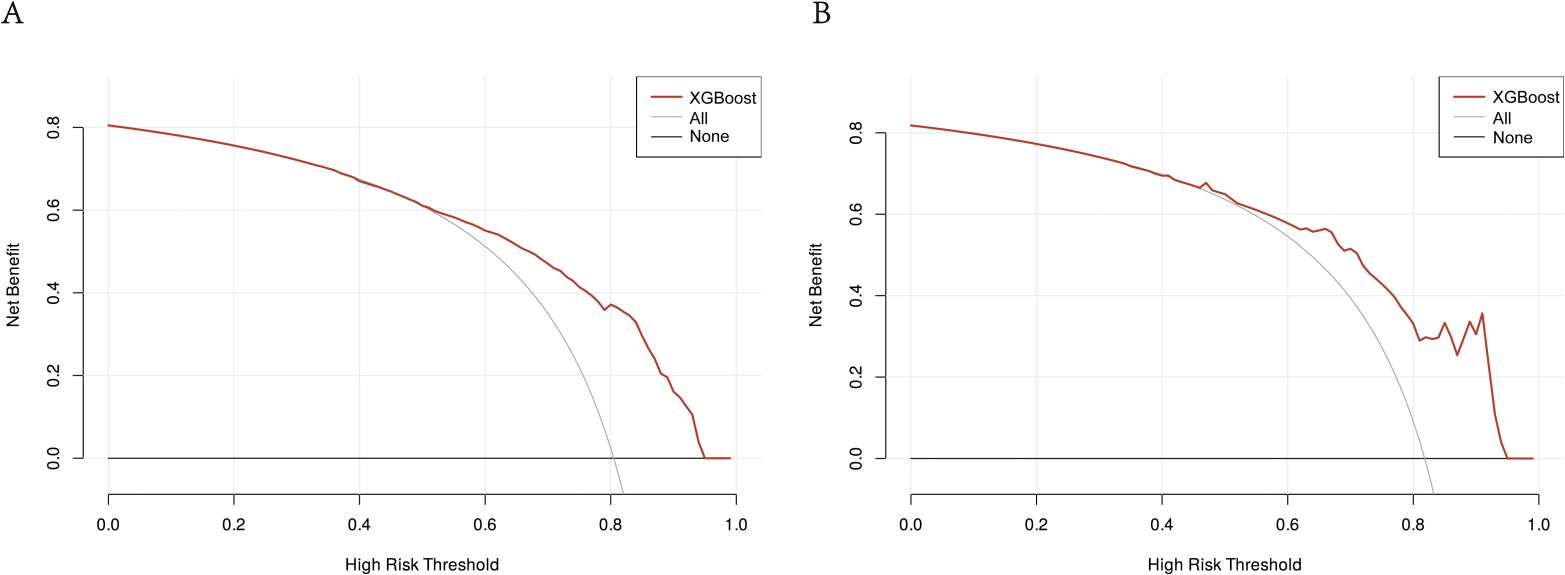
DCA of the XGBoost model: **(A)** DCA for the training group. **(B)** DCA for the validation group. The clinical decision curves for both the training and validation groups demonstrate that the model outperforms the “treat all” and “treat none” strategies across a broad range of threshold probabilities. DCA, Decision curve analysis; XGBoost, Extreme Gradient Boosting.

In the training cohort and validation cohort, the Brier values were 0.119 (95% CI, 0.088-0.152) and 0.122 (95% CI, 0.092-0.156), respectively (**Figure 4**). To further assess the efficacy of the XGBoost model, clinical decision curves were constructed to appraise its clinical utility. The clinical decision curves for both the training and validation sets demonstrate that the model’s performance surpasses the “treat all” and “treat none” strategies across a broad range of threshold probabilities (**Figure 5**). This demonstrates the remarkable clinical applicability of the models, confirming their effectiveness in facilitating clinical decision-making.

### 3.4 The significance of features in ML models

**Figure 6:**
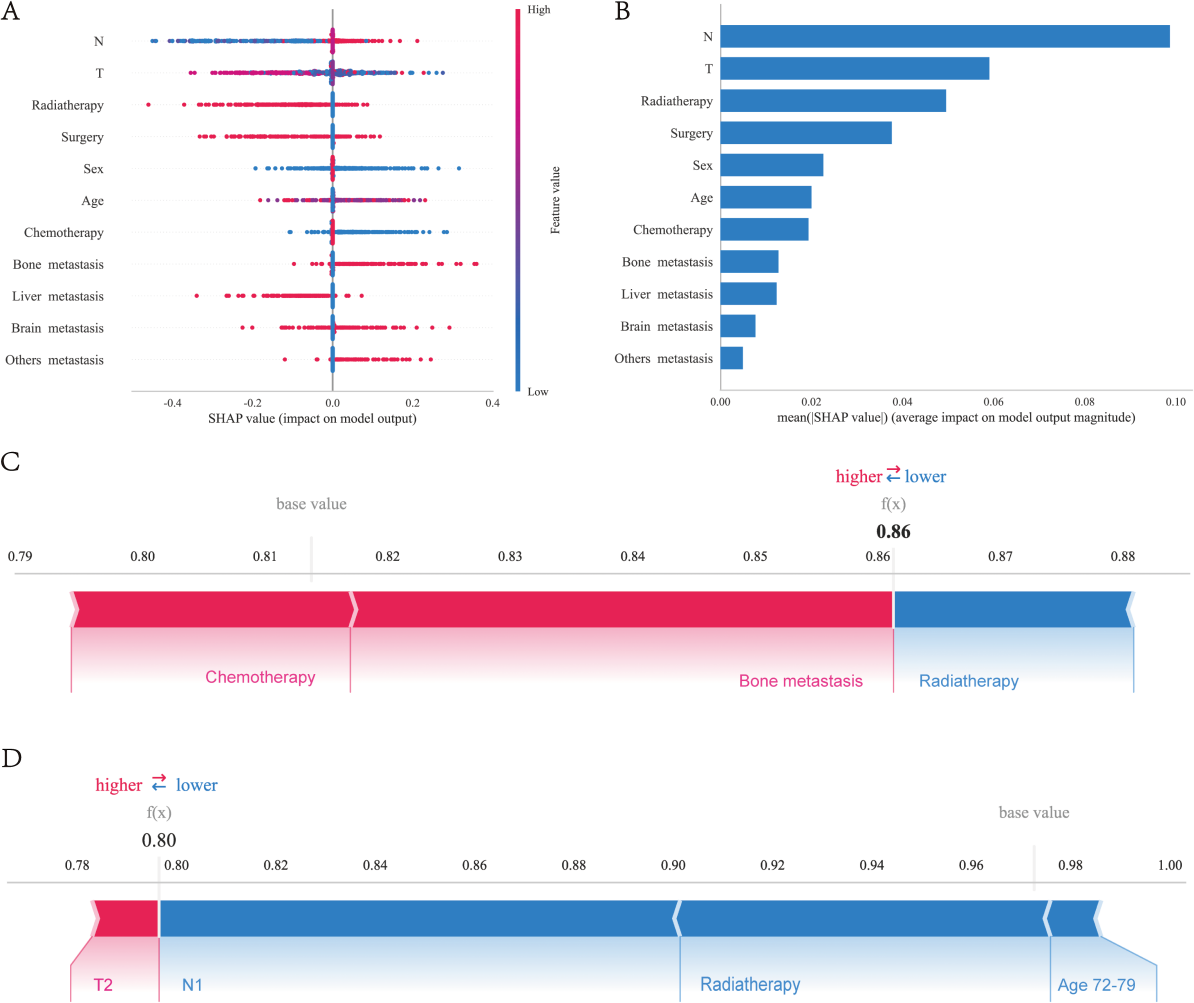
Interpretability analysis of XGBoost models: **(A)** SHAP dendrogram of features.X-axis: SHAP values, which indicate the impact of each feature on the model’s output. Positive values increase the predicted value, while negative values decrease it.Y-axis: Different features. Color: Represents the value of the feature, with red indicating high values and blue indicating low values. Dots: Each dot represents the SHAP value for a specific sample. **(B)** Importance ranking plot of features.X-axis: The mean SHAP value of each feature, indicating the average impact of the feature on the model’s output.Y-axis: The names of the features. Bars: The length of each bar represents the importance of the feature, with longer bars indicating a greater impact on the model’s output. Interpretability analysis of 2 independent samples:**(C)** Sample A. **(D)** Sample B.

We utilized the SHAP diagram to vividly illustrate the importance of each feature in our mode (**Figure 6A**). The left-hand side of the graph enumerates all the features. The x-axis depicts SHAP values, which quantify the influence exerted by each feature on the model’s output. Positive SHAP values signify an increase in the predicted outcome due to the feature, whereas negative values denote a decrease. The color gradient, spanning from blue to red, signifies the range of feature values from low to high. Specifically, in the case of “N stage”, blue dots symbolize lower stages, while red dots represent higher stages. The scatter of dots for each feature visualizes the varying impacts of that feature on the model’s predictions across its value spectrum. Dots situated farther from the origin (0) indicate a more pronounced effect of the feature on the model’s output. **Figure 6B** displays the mean absolute SHAP values, highlighting the average impact of each feature on the model’s output. N stage is the most critical feature, followed by T stage and Radiotherapy, which also significantly influence survival prognosis. Surgery, sex, and age also play notable roles, but their impact diminishes in order.

### 3.5 Individualized model interpretability using SHAP

To elucidate the model’s decision-making process at an individual level, we performed an in-depth interpretability analysis on two exemplary samples, as depicted. In the first SHAP sample plot (**Figure 6C**), three features were presented to illustrate their contributions to the model’s predictions: chemotherapy and bone metastasis have a positive impact on the model’s predictions, while radiotherapy has a negative impact on the model’s predicted value in this sample. It can also be observed that the factor of bone metastasis has the highest influence on the model’s output value. In the second SHAP sample plot (**Figure 6D**), it has been observed that age between 77-79 years, radiotherapy, and stage N0 have a negative impact on the model’s predicted values, while stage T2 has a positive impact, albeit a small one. Additionally, stage N0 has the greatest influence on the model’s final output values.

### 3.6 Web-based predictive tools

In order to enhance the clinical utility of the predictive model, we have constructed an interpretable web-based clinical prediction tool for forecasting patients’ 1-year survival probability (http://www.xsmartanalysis.com/model/list/predict/model/html?mid=21255&symbol=2173ECSk6aW9dh146485).

## 4 Discussion

C-SCLC is an exceptionally lethal and heterogeneous subtype of lung cancer that remains relatively uncommon (19, 20). SCLC comprises 15% of all lung cancer diagnoses (21), with C-SCLC accounting for 5%~28% of SCLC cases (2). Nevertheless, advancements in diagnostic techniques may be leading to an increase in the incidence of combined small cell lung cancer (22). Despite the efforts of scholars from all fields, the prognosis for C-SCLC patients remains dire, highlighting the pressing need for refined prognostic models (23–25).

In the study by Yang et al. (15), researchers developed a nomogram model for predicting the prognosis of patients with C-SCLC based on clinical features including age, sex, TNM stage, surgery, and chemotherapy (15). However, this model did not undergo external validation. In contrast, our study further refined tumor metastasis status, demonstrating its significant contribution within the model. Additionally, we performed external validation using an independent cohort of Chinese patients to assess the model’s predictive performance across different racial groups. We also employed the SHAP method to interpret the ‘black-box’ model, thereby enhancing both its credibility and clinical utility. Finally, we developed a web-based tool to facilitate the practical application of this model in clinical settings.

At present, the treatment of C-SCLC is based on the National Comprehensive Cancer Network (NCCN) guidelines for SCLC, which do not further refine the standard treatment protocols for this specific type of cancer (26, 27). In prior research, the effect of radiotherapy on patients with C-SCLC remained uncertain (2). To date, no large-scale prospective clinical trials have been conducted to evaluate the role of thoracic radiotherapy in the treatment of patients with C-SCLC. In our study, multivariable Cox regression analysis showed that C-SCLC patients receiving radiotherapy had a better prognosis, with an HR of 0.659 (95% CI: 0.552-0.787, p<0.001). This indicates that radiotherapy is an independent prognostic factor for all-cause mortality. Surgical intervention is gaining increasing attention in SCLC patients and has also sparked significant interest in the context of composite carcinoma (3, 28). Our study confirmed that surgery is also an independent prognostic factor for all-cause mortality in C-SCLC patients, with a hazard ratio (HR) of 0.415, a 95% confidence interval (CI) of 0.328-0.526, and a p-value of less than 0.001.

In reality, at the time of initial diagnosis, the vast majority of C-SCLC patients are already in the advanced stage and have lost the opportunity for surgery. The reason may be that most patients are diagnosed through surgical pathology. In C-SCLC, the histological component of SCLC accounts for the vast majority of the tumor tissue, and the diagnostic rates of CT-guided lung biopsy, bronchoscopy biopsy, or cytological examination are relatively low. The reason is that ordinary biopsy or cytological examination yields a small amount of tissue sample, which is prone to missed diagnosis or misdiagnosis, and cannot accurately determine the tissue type of the tumor (29). Therefore, we believe that for limited-stage SCLC patients, the indications for surgery can be appropriately expanded according to the situation. This can avoid the possibility of misdiagnosis due to insufficient biopsy samples at the initial examination and can also confirm whether there is transformation to N-SCLC (Non-Small Cell Lung Cancer).

In terms of model interpretability, we choose the SHAP technique to address the “black box” issue of machine learning models, calculating the incremental effect of each feature on the model output and leveraging additive explanation models (30). The reason is that, unlike LIME (Local Interpretable Model-Agnostic Explanations) which focuses on analyzing individual predictions and their causes and is suitable for personalized interventions, SHAP has more advantages in understanding overall feature importance, identifying consistent patterns, determining the priority of risk factors, and guiding group-level interventions (31, 32).

Our research shows that surgery and radiotherapy, along with T and N staging, significantly impact model output. An interactive web-based prediction tool was developed to support clinical decision-making. For a C-SCLC patient who hasn’t had radiotherapy or surgery, we can input their clinical characteristics into the tool to get a prediction result. By assuming they receive radiotherapy or surgery and getting another prediction result, we can clearly see the treatment plan’s impact on survival probability. This provides a strong basis for clinical decision-making. For confused or worried patients, doctors can show them the SHAP analysis chart to explain the effects of various factors on prognosis and the reasons for the chosen treatment plan. After understanding the impact of different treatment plans on survival probability, patients can discuss the most appropriate treatment plan with doctors based on their own values, quality of life, and expected lifespan. This patient-participatory treatment decision-making model will improve patients’ treatment compliance and their treatment experience and psychological state. The importance of interpretability in clinical applications of ML models cannot be overemphasized, as it fosters trust and facilitates the integration of these models into clinical practice (33, 34). These findings corroborate with prior research that has underscored the potential of ML in predicting survival outcomes across various cancer types (33, 35, 36).

As far as we know, this is the largest study that has applied machine learning to the prognostics of C-SCLC. Nevertheless, there are several significant limitations in this study. Firstly, the clinical pathological diagnosis in the SEER database cannot determine the existence of composite components, which have a significant impact on the treatment plan selection and prognosis of C-SCLC patients (37). Secondly, the SEER database lacks information on tumor markers, smoking history, genetic history, gene mutation information, family history, as well as specific chemotherapy and radiotherapy regimens, molecular targeted therapy, and immunotherapy. Our study also did not involve the impact of these factors on the prognosis of C-SCLC patients. Screening these factors and incorporating them into modeling is expected to yield a clinical prediction model with higher discrimination and accuracy. Lastly, when this machine learning model is validated in a larger external cohort, the validation results may change. In the future, large-scale prospective multicenter cohorts are needed to verify and optimize this model.

## 5 Conclusion

In this study, we innovatively applied the XGBoost-based machine learning model to develop a survival prediction system for C-SCLC patients. By leveraging demographic characteristics and pathological indicators obtained from the SEER database, we prioritized the significance of various features. The model’s performance was comprehensively assessed using ROC curves, precision curves, calibration plots, and decision curves. Additionally, an external validation cohort was established to further corroborate the model’s reliability. Ultimately, we created an interactive web-based prediction tool to support clinical decision-making.

## Data Availability

The raw data supporting the conclusions of this article will be made available by the authors, without undue reservation.
